# Calcium Absorption in Infants and Small Children: Methods of Determination and Recent Findings 

**DOI:** 10.3390/nu2040474

**Published:** 2010-04-06

**Authors:** Steven A. Abrams

**Affiliations:** Department of Agriculture, Agricultural Research Service, Children's Nutrition Research Center, Department of Pediatrics, Baylor College of Medicine and Texas Children's Hospital, Houston, Texas 77030, USA; Email: sabrams@bcm.edu

**Keywords:** calcium absorption, infant nutrition, bone mineral content

## Abstract

Determining calcium bioavailability is important in establishing dietary calcium requirements. In infants and small children, previously conducted mass balance studies have largely been replaced by stable isotope-based studies. The ability to assess calcium absorption using a relatively short 24-hour urine collection without the need for multiple blood samples or fecal collections is a major advantage to this technique. The results of these studies have demonstrated relatively small differences in calcium absorption efficiency between human milk and currently available cow milk-based infant formulas. In older children with a calcium intake typical of Western diets, calcium absorption is adequate to meet bone mineral accretion requirements.

## 1. Introduction: Assessment of Calcium Absorption in Children

The determination of calcium requirements in children has largely been done through the evaluation of the amount of calcium absorbed and retained (factorial methodology). There have been few controlled calcium supplementation trials in children <9 years of age. Few of these trials have provided long-term follow-up to determine if the short-term benefits observed were maintained after stopping the supplementation. In addition, very few studies have evaluated outcomes from calcium supplementation other than those related to bone mineralization in infants and children. Therefore, it is important to accurately assess both calcium intake and calcium absorption in order to develop rational values for a factorial determination of calcium requirements.

Evaluating calcium status in infants and small children poses several unique challenges. First, in most circumstances, calcium intake is difficult to accurately measure in breast-fed infants. Second, dietary surveys, such as the NHANES survey related to calcium intakes in older infants usually are principally sampling formula-fed infants who are the predominant group of older infants in the United States. Relatively few data are available related to older, primarily breast-fed infants who may have a mixed diet but are not receiving any infant formula or cow milk [1]. 

Assessing calcium absorption in children is technically problematic due to cost and compliance issues with the historically used metabolic balance technique. Metabolic balance studies, in which dietary intake and fecal and urine samples are collected for at least 3 days (5 to 7 days in older children) that were common prior to the 1960’s are virtually impossible to perform presently in small children. A prolonged urinary and fecal collection, either using collection devices (bags) or metabolic beds is almost inconceivable for full-term infants or small children at the present time. Families do not have this length of time to stay with their child in an inpatient setting and the costs of hospitalization and nursing care for this length of time is prohibitive. For breast-fed infants this is made even more challenging by the difficulty of accurately assessing calcium intake during normal breast-feeding. 

Two alternative techniques can be applied to the assessment of calcium absorption and accretion in infants and small children. The first of these is the dual-tracer stable isotope technique [2,3]. In this technique, one non-radioactive isotope of calcium is given orally and a second isotope is given intravenously. Isotopes used are usually 42Ca, 44Ca, or 46Ca, although both 43Ca and 48Ca can also be used. The choice of isotopes and dosing is based on the analytical method to be used in the study, not the subject population. Some mass spectrometric techniques are more readily able to analyze different isotopes and therefore discussion with the planned analytical laboratory is necessary prior to beginning a study. Calcium stable isotopes are safe for use in children of all ages. We have conducted several thousand studies in our laboratory with no complications of any form having occurred.

Typically, the isotope doses are mixed in milk or infant formula 12-24 hours before administration in order to allow for tracer equilibration [2,3]. Fractional calcium absorption (absorption efficiency) is calculated from the relative recovery of the oral *versus* the intravenous tracer during the 24 hours after isotope administration. The benefits of this approach are that only 24 hours of a monitored stay are needed and that fecal collections are not needed. Major disadvantages are that an intravenous line needs to be started (although only for about 1 minute) and that a 24 hours urine collection is needed. The infusion must be done using appropriate sterile technique. Isotopes given intravenously must be tested prior to use by an appropriate laboratory for sterility and pyrogenicity. A further limitation is that this method only calculates dietary absorption and does not account for any secretory losses of calcium. These need to be estimated to obtain a measure of calcium accretion (balance) [2]. However, adequate data are available in the literature for this estimation and secretion is not a primary regulatory site for healthy infants and small children [4]. 

The dual tracer technique has become accepted in both adults and children as the standard for assessing calcium absorption from a single meal. In infants, because each meal is relatively similar, tracing a single meal should accurately reflect the overall daily calcium absorption. In older children, it is important to balance, as much as possible, the calcium intake during the study day. In general, diets are provided for the subject during the day of the isotope study and some effort is made to assure that the dietary calcium intake is not highly variable during the 7 to 10 days before the study. Some groups will provide all the meals during this time, while others will provide detailed guidance on the diet to be given at home. It is not practical to hospitalize the subjects for this length of time to ensure dietary compliance.

In rapidly growing children, the rate of change in total body bone mineral content can also be used to estimate long-term dietary calcium retention. In this approach two DXA measurements of total body bone mineral content are performed and the difference between the two divided by the interval, adjusted for the proportion of bone mineral that is calcium is used to calculate an average daily calcium accretion (retention). Benefits to this approach are that no body fluid or blood collections are needed [5]. Disadvantages are the difficulty of doing DXA in small infants. This consists primarily of the challenge of keeping infants from moving during the DXA scan and thus avoiding movement artifacts. A more substantial limitation is that changes in total body calcium as assessed by DXA occur slowly and are measured over months, not days or weeks. It is typical that even those measurements performed in infants, are separated by at least 3 and often 6 months. Therefore, what is measured integrates the changes in calcium absorption over the multiple months of the study and is not a sensitive method to assess differences related to type of infant feeding or any short-term individual dietary manipulation.

## 2. Results and Discussion

### 2.1. Calcium Absorption from Infant Formula Compared to Human Milk

The available metabolic data relating to calcium absorption in infants in the first months of life were summarized by Nelson and Fomon in 1993 [6]. Not all of the data compiled in that chapter had been previously published or compiled and there are no similar comprehensive sources of mass balance data in infants. Key aspects of this publication were that calcium absorption efficiency was about 60% from human milk and about 40% from infant formulas during the first four months of life. This led to a calculated net calcium retention that was similar between formula and human milk-fed infants of about 160-170 mg/day. However, the calculated calcium intake of 327 mg/day in the first 4 months of life by breast-fed infants in these data [6] is far above the amount determined in more recent studies of breast-fed infants. Because of this high intake, the net calcium retention [6] from both breast milk and infant formula are far above those believed to occur based on total body bone mineral content data and more recent isotope based studies [6,7,8,9,10]. Similarly, data from Nelson and Fomon [6] from both formula-fed and whole cow milk-fed infants in the second six months of life show mean calcium intakes of 600-900 mg/d. These intakes are far above those achieved by primarily breast-fed infants [1] and therefore it is not surprising that the calcium retention of 240-280 mg/day in 6 to 12 month infants calculated by Fomon and Nelson is much higher than typical values for calcium retention in infants derived from DXA data that included a substantial number of breast-fed infants (see below).

**Figure 1:**
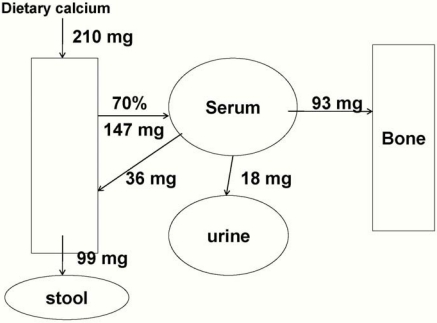
Calcium balance in a typical 6 kg exclusively breast-fed infant. Net calcium retention of 93 mg/day is shown as going to bone.

Using DXA, Butte [7] found that 119 mg calcium is accreted daily during the first year-about 110 mg daily in girls and 130 mg daily in boys. Specker [8] reported a slightly higher of about 140 mg daily in a group of primarily formula-fed infants. It is impossible to determine the effects of solid food or formula intake on these data or identify the exact rate of calcium accretion specifically during the first 6 months of life, but given a usual calcium intake from solids after 6 months of age of about 60-80 mg/day [1], an average retention of 100 mg daily calcium accretion during exclusive breast-feeding and 140 mg daily on mixed feedings can be estimated. Figure 1 shows a model system with the intake, losses and net calcium retention (93 mg/day, shown as going to bone) of a typical human milk-fed baby who is not receiving any solid food. The values for intake and absorption are based on estimates from mixed-fed infants as well as the older balance data [6,10].

Additional data related to the absorption of calcium from infant formula comes from a study using partially hydrolyzed formulas with and without lactose [9]. The absorption percentage from the lactose-containing formula was 66%. This does not account for about 5% likely lost via secretory losses. A net absorption of approximately 60% is consistent with data from human milk fed infants both as published by Nelson and Fomon [6] and as published in older breast-fed infants [10]. This result suggests that it is not necessarily the case that calcium absorption efficiency from human milk is substantially greater than that from currently marketed infant formulas, although further data are needed for infants fed non-partially hydrolyzed formula. Since, by regulatory statute in the United States and tradition in many countries, the concentration of calcium in all infant formulas markedly exceeds that of human milk, it is impossible to truly know if there is an intrinsic higher bioavailability of calcium in human milk compared to formula. Even if a difference does exist, the difference is likely to be small and may not have important biological significance.

In summary, it is not clear that the inherent bioavailability of calcium in currently used infant formulas is substantially different than from human milk. Because the intake of calcium is much higher from all infant formulas marketed in the United States, the net calcium absorption and retention is higher in formula-fed infants. Whether this is beneficial or harmful is unknown.

### 2.2. Post-infancy Calcium Absorption: Ages 1 to 3 Years

There are very few published data related to calcium intake and absorption in children 1 to 3 years of age. This age period does not appear to be a time of rapid bone mineral accrual, especially when considered on a body weight basis. However, cases of clinical rickets are widely reported during the second year of life [11]. Some of these cases are likely associated with the combined effects of low vitamin D status and low calcium intake rather than being entirely due to vitamin D deficiency. 

**Figure 2:**
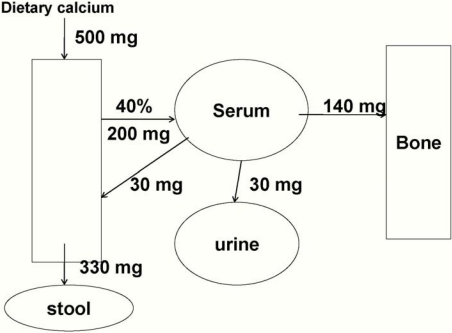
Calcium balance in a typical 20 kg toddler. Net calcium retention of 140 mg/day is shown as going to bone.

Mean calcium intake of about 800 mg/day is reported in this age group [1] with values at the 5th percentile of about 400 mg/day. Limited DXA data in this age group suggest that the accretion of calcium is similar to that in the first year of life, about 100-150 mg/day [1,12]. Recent stable isotope balance studies in this population demonstrated that intakes of 500-600 mg/day would be adequate to achieve this target, with the higher intake levels meeting the needs of nearly all individuals [13] (Figure 2). An interesting question is the ability of children this age to adapt to diets extremely low in calcium intake. A series of studies in Nigeria has shown that calcium absorption is as high as 60-80% in children receiving very low calcium intakes (about 200 mg/d). [14,15,16]. This remarkable ability to adapt to extremely low calcium intakes leads to an ability to avoid rickets in most, but not all children with very low calcium intakes.

In considering the dietary intake *versus* requirements, it is clear that the majority of the population easily meets its intake requirement [1]. It is likely that populations that do not meet the 500-600 mg/day intake recommended are primarily those that use little or no cow milk-derived products and few fortified soy milk products of supplements.

### 2.3. Calcium Absorption in Older Pre-Pubertal Children (4 to 8 years)

By the age of 4 years, diets are more representative of adult patterns and assessment of calcium intake usually uses dietary recalls or weighed foods. Limitations in these approaches as would apply to all age groups are present for children as well. Several small studies have looked at calcium absorption in this age group. This age group is likely to be a relatively low risk one for significant calcium deficiency. The rapid bone growth of puberty has not begun and most children continue to have relatively high intakes of dairy products providing the majority of their calcium intakes. Special attention should be paid to strict vegetarian children or those with lactose intolerance who avoid most or all dairy products.

In 1999, it was reported that intakes of about 1100 mg/d led to a net calcium retention of about 200 mg/d [17]. In 2001, it was reported that intakes of about 900 mg/d led to calcium retention of about 172 mg/d [18] and that intakes of about 700 mg/d let to retentions of about 120 mg/day. There is a substantial variability in the amount of skeletal calcium accreted in this age range, but values of 140-160 mg/day are reasonable and would be met with an average intake of about 800 mg/day. 

## 3. Conclusions

In infancy, based on calcium intakes that vary from as low as 200 mg/day in exclusively breast-fed infants in the early months of life to up to 900 mg/day in formula-fed infants receiving some solids nearly at a year of age, it is likely that calcium absorption depends primarily on intake, not on human *vs*. formula source and that absorption fraction ranges from about 60% at the low intake end to about 30% at the high intake end. In older children, where intake is more likely to range from 400 to about 1000 mg/day, absorption fraction likely ranges from about 40% at low intake levels to about 25% at the higher levels. These ranges lead to, in most infants an average net accretion between about 80-160 mg/day during the first 8 years of life. There is no evidence for targeting any one accretion value at any given age nor is their evidence of harm from higher intakes.
